# Ferroptosis Suppression by the M^6^A Reader IGF2BP1 Underlies Gemcitabine Resistance in Pancreatic Ductal Adenocarcinoma

**DOI:** 10.1002/advs.77048

**Published:** 2026-08-10

**Authors:** Ying‐Qin Zhu, Yue Huang, Jing‐Yuan Ye, Si‐Yuan Lu, Zhi‐De Liu, Ming‐Jian Ma, De‐Liang Fang, Zi‐Yi Zhao, Jin‐Zhao Xie, Jiao Chen, Xiao‐Yu Yin

**Affiliations:** ^1^ Department of Pancreato‐Biliary Surgery The First Affiliated Hospital of Sun Yat‐sen University Guangzhou Guangdong China; ^2^ Department of Medical Oncology The Second Affiliated Hospital of Guangzhou Medical University Guangzhou Medical University Guangzhou Guangdong China; ^3^ Department of Hepatobiliary Pancreatic Tumor Center Chongqing University Cancer Hospital Chongqing China

**Keywords:** ferroptosis, FTH1, IGF2BP1, N6‐methyladenosine, pancreatic ductal adenocarcinoma, stress granule

## Abstract

**Aims:**

Pancreatic ductal adenocarcinoma (PDAC) is a lethal malignancy with poor prognosis, and gemcitabine‐based chemotherapy remains the standard first‐line treatment. However, the rapid emergence of chemoresistance limits its efficacy. Elucidating the molecular mechanisms underlying gemcitabine resistance is critical for improving therapeutic outcomes.

**Approaches & Results:**

By integrating transcriptomic profiling of gemcitabine‐resistant PDAC cells, patient‐derived xenografts (PDXs), and pretreatment clinical biopsy specimens, we identified the m^6^A reader IGF2BP1 as a key determinant of both intrinsic and acquired gemcitabine resistance. Mechanistically, IGF2BP1 directly binds m^6^A‐modified FTH1 mRNA and cooperates with G3BP1‐mediated stress granule sequestration to enhance FTH1 transcript stability under chemotherapeutic stress. The stabilized FTH1 maintains intracellular iron homeostasis, limits lipid peroxidation, and suppresses ferroptosis, thereby conferring chemoresistance. Pharmacological inhibition of IGF2BP1 with BTYNB partially disrupted this regulatory axis, restored ferroptotic susceptibility, and synergistically enhanced gemcitabine efficacy in resistant PDX models without overt toxicity.

**Conclusions:**

Collectively, this study uncovers a previously unrecognized epitranscriptomic mechanism linking m^6^A‐dependent RNA stabilization, stress granule dynamics, and ferroptosis suppression in PDAC. By identifying IGF2BP1 as a potential biomarker of gemcitabine response and a therapeutically actionable target, these findings provide a preclinical rationale for IGF2BP1‐targeted combination strategies to overcome gemcitabine resistance in PDAC.

## Graphical abstract

IGF2BP1 promotes gemcitabine resistance in pancreatic cancer by stabilizing m^6^A‐modified FTH1 transcripts and protecting them in stress granules, thereby suppressing ferroptosis. Pharmacological inhibition of IGF2BP1 disrupts this protective pathway, restores ferroptotic sensitivity, and enhances gemcitabine efficacy in resistant tumors. (This schematic was created with BioRender.com. Agreement number: WC290LWCFS).

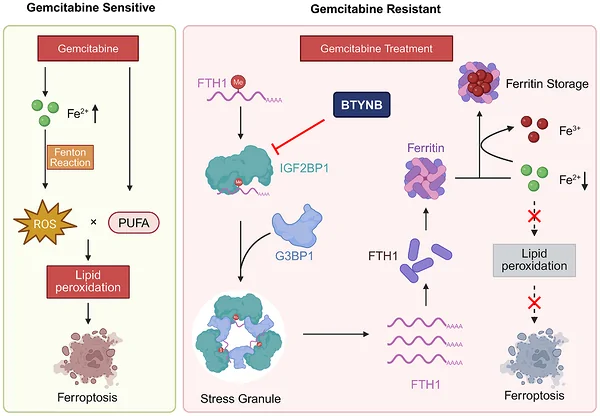

**Abbreviations**
CI: combination index
Co‐IP: Co‐immunoprecipitation
DFO: deferoxamine mesylate
FTH1: Ferritin Heavy Chain 1
G3BP1: Ras‐GTPase‐activating protein (GAP)‐binding protein 1
GSEA: Gene Set Enrichment Analysis
IGF2BP1: Insulin‐like growth factor 2 mRNA‐binding protein 1
IGV: Integrative genomics viewer
IHC: immunohistochemistry
LC‐MS/MS: liquid chromatography‐tandem mass spectrometry
LPO: lipid peroxide
m^6^A: N6‐methyladenosine
meRIP: methylated RNA immunoprecipitation
PD: progressive disease
PDAC: Pancreatic ductal adenocarcinoma;
PDX: patient‐derived xenograft
PR: partial response
RECIST1.1: Response Evaluation Criteria in Solid Tumors
RI: Resistance Index
ROS: reactive oxygen species
SD: Stable Disease
SGs: Stress granules

## 1 Introduction

Pancreatic ductal adenocarcinoma (PDAC) is an aggressive malignancy with a rapidly rising incidence and a five‐year survival rate below 10% [1]. Over 80% of patients present with locally advanced or metastatic disease, precluding curative surgery and rendering chemotherapy the primary treatment modality [2]. Although gemcitabine‐based combination regimens are the recommended first‐line therapy, they achieve response rates below 20% [3], with relapse or progression in over 74% of patients [4]. Clarifying the mechanisms underlying gemcitabine resistance and developing novel combination strategies are essential for improving patient outcomes.

N6‐methyladenosine (m^6^A), a dynamic RNA modification governing transcript stability and translation [5], has emerged as a critical epigenetic regulator of tumor biology. Cellular m^6^A modifications are dynamically controlled by methyltransferases (“writers”) and demethylases (“erasers”), while m^6^A “readers”—including the YTHD family and IGF2BP family—direct the fate of modified RNAs. IGF2BP1 (Insulin‐like growth factor 2 mRNA‐binding protein 1), a recently identified m^6^A reader, plays a key role in tumorigenesis and progression across malignancies. Early studies revealed that IGF2BP1 enhances tumor cell proliferation by activating β‐catenin signaling through the stabilization of βTrCP1 and c‐MYC mRNAs [6, 7]. Subsequent research has confirmed that its RNA‐stabilizing function is critically dependent on m^6^A modification [8]. Since then, accumulating evidence has demonstrated that elevated IGF2BP1 drives malignancy in colorectal cancer [9], ovarian cancer [10], osteosarcoma [11], and gastric cancer [12]. However, its role in PDAC remains insufficiently defined, particularly regarding how it integrates with m^6^A‐dependent RNA stabilization to promote chemoresistance.

Stress granules (SGs) effectively prevent aberrant protein translation under stress conditions by safeguarding the free mRNAs of stress‐related proteins, thereby enhancing cellular resilience to adverse environmental stimuli and promoting cell survival [13]. G3BP1 serves as a central nucleating factor in SG assembly. Under physiological conditions, G3BP1 is diffusely distributed throughout the cytoplasm. However, in response to stressors such as hypoxia or chemotherapy, polysome disassembly releases large quantities of mRNA, prompting G3BP1 to aggregate and initiate SG formation. Within these granules, G3BP1 interacts with numerous RNA‐binding proteins and mRNAs to form dynamic protein‐RNA condensates [14].

Ferroptosis is an iron‐dependent form of regulated cell death that is mechanistically distinct from apoptosis, necrosis, and autophagy. It plays a pivotal role in tumor progression, metastasis, and chemoresistance, characterized by the excessive accumulation of intracellular ferrous ions (Fe^2^^+^), glutathione depletion, and lipid peroxides. A hallmark event in ferroptosis is the aberrant increase of free Fe^2^^+^, which catalyzes the formation of reactive oxygen species (ROS), including hydroxyl radicals, thereby driving membrane lipid peroxidation and ultimately causing cell death. The accumulation and intracellular release of iron are key determinants of ferroptosis, while lipid peroxidation represents its final phenotypic outcome [15]. Ferritin, composed of the heavy (FTH1) and light (FTL) subunits, maintains intracellular iron homeostasis by sequestering labile Fe^2^^+^. In particular, FTH1 mitigates ferroptosis by oxidizing Fe^2^^+^ to the inert Fe^3^^+^ form for safe storage, and its overexpression markedly suppresses ferroptosis [16]. Recent studies indicate FTH1‐mediated iron sequestration as a key adaptive mechanism in cancer cells.

We established gemcitabine‐resistant pancreatic cancer cell and PDX models. Integrated transcriptomic, MeRIP‐seq, and RIP‐seq analyses identified IGF2BP1 as a key resistance‐associated m^6^A reader in gemcitabine‐resistant PDAC models. Mechanistically, IGF2BP1 directly binds m^6^A‐modified FTH1 transcripts and cooperates with G3BP1‐mediated stress granule sequestration to protect FTH1 mRNA from degradation, thereby preserving iron homeostasis and suppressing ferroptosis. Pharmacological inhibition of IGF2BP1 restored ferroptotic sensitivity and enhanced gemcitabine efficacy, highlighting a therapeutically actionable epitranscriptomic vulnerability in PDAC.

## 2 Materials and Methods

### 2.1 Patients and Specimens

Human PDAC samples were collected at the First Affiliated Hospital of Sun Yat‐sen University (2016‐2020). The follow‐up rate was 100% (last follow‐up: November 10, 2024). A total of 45 pre‐chemotherapy needle biopsy specimens were obtained from patients who met the inclusion criteria, including pathological confirmation of PDAC and no prior radiotherapy or chemotherapy. All patients received gemcitabine‐based chemotherapy and were followed for clinical outcomes. Per Response Evaluation Criteria in Solid Tumors (RECIST1.1) [17], patients were categorized as gemcitabine‐resistant (progressive disease, PD, *n* = 16), gemcitabine‐sensitive (partial response, PR, *n* = 4), or stable disease (stable disease, SD, *n* = 25). Additionally, 10 fresh surgical specimens (2018‐2020) were prospectively collected for patient‐derived xenograft (PDX) model establishment.

### 2.2 Gemcitabine‐Resistant Cell Lines

BxPC‐3 and CFPAC‐1 cells (Chinese Academy of Sciences Cell Bank, Shanghai, China) were seeded in 96‐well plates and treated with escalating gemcitabine concentrations (10 nm to 1 µ) for 48 h. CellTiter‐Glo assays determined IC_50_ values. Resistant lines (BxPC‐3/GR, CFPAC‐1/GR) were then established by continuous 6‐month exposure to incrementally increased gemcitabine doses (10 nm–1µm, doubled every 2 weeks), with ≥10‐fold IC_50_ elevation confirmed.

### 2.3 Gemcitabine‐Resistant PDX Models

Patient‐derived tumor fragments (3 mm^3^) were subcutaneously engrafted into 5‐week B‐NDG mice (Biocytogen, Beijing, China). Tumor volume (0.5 × length × width^2^) was monitored, and tumors were passaged upon reaching 1 cm^3^. Then the tumors were excised, subdivided, and re‐implanted to establish the first‐generation (F1) PDX. F1 tumor‐bearing mice were randomized to PBS or gemcitabine (25 mg/kg, i.p., biweekly) treatment. Serial passaging through three generations (F1‐F3) established gemcitabine‐resistant PDX models, with selection requiring Resistance Index (RI = [drug‐treated tumor growth rate]/[control growth rate] × 100%) >150%.

### 2.4 Animal Experiments

Genetic intervention: IGF2BP1‐modified BxPC‐3/GR or CFPAC‐1/GR cells were implanted subcutaneously in 4‐week BALB/c nude mice. Gemcitabine treatment (25 mg/kg, i.p., biweekly) commenced at tumor volume = 50 mm^3^. Tumors (volume = length×width^2^/2) and weights were monitored biweekly and weekly, respectively. After 4 weeks, tumors were harvested for Ki67 IHC analysis.

Drug combination: gemcitabine‐resistant PDX tumors were implanted and randomized to: PBS, BTYNB (10 mg/kg, oral, daily), gemcitabine (25 mg/kg, i.p., biweekly), or combination. Tumor/weight measurements followed the above schedule. Endpoint analyses (day 21) included hematology, hepatic/renal function, and Ki67 IHC.

### 2.5 Statistical Analysis

All experiments were performed with at least three independent biological replicates unless otherwise stated. Data are presented as mean ± standard deviation (SD). For comparisons between two groups, statistical significance was evaluated using a two‐tailed unpaired Student's t‐test. For comparisons among multiple groups, one‐way analysis of variance (ANOVA) followed by appropriate post hoc tests (Tukey's or Dunnett's test, as specified in each figure legend) was applied. or experiments involving tumor growth curves over time, repeated‐measures ANOVA was used to evaluate differences among groups. Correlations by Spearman coefficient. Drug combination effects were evaluated via Chou‐Talalay combination index (CI). Significance: ^*^ *p* < 0.05; ^**^ *p*< 0.01; ^***^ *p*< 0.001; ns, not significant. Exact n values (biological replicates or sample size) are indicated in each figure legend.

## 3 Results

### 3.1 IGF2BP1 is Upregulated in Gemcitabine‐Resistant PDAC and Correlates With Poor Clinical Outcome

To investigate the mechanisms underlying gemcitabine resistance in PDAC, we established gemcitabine‐resistant PDAC cell lines (BxPC‐3/GR and CFPAC‐1/GR) (Figure 1A), which showed markedly increased IC_50_ values compared with parental cells (Figure S1A,B). Transcriptomic profiling revealed distinct expression patterns between resistant and parental lines (Figure 1B). Integrative analysis of these data with TCGA, GTEx, and GEO (GSE154909) datasets identified genes consistently upregulated in PDAC tumors, gemcitabine‐resistant cells, and resistant PDXs. Notably, the m^6^A reader IGF2BP1 was found to be one of the commonly upregulated genes (Figure 1C). Clinically, elevated IGF2BP1 expression in PDAC was significantly correlated with poorer prognosis (Figure S1C,D). Crucially, immunofluorescence analysis of pre‐treatment biopsies from PDAC patients revealed higher baseline IGF2BP1 expression in tumors that were inherently refractory to gemcitabine‐based chemotherapy (Figure 1D). Functionally, PDXs with high IGF2BP1 levels exhibited reduced gemcitabine sensitivity in vivo (Figure 1E,F). To validate these findings, we generated a complementary gemcitabine‐resistant PDX model through serial passaging under continuous gemcitabine selection (Figure 1G). Sequential PDX samples collected across generations were subjected to RNA sequencing and immunohistochemical analysis. Genes exhibiting progressively increased expression during resistance acquisition were enriched in pathways related to “*mRNA binding*” and “*N6−methyladenosine−containing RNA reader activity*” (Figure 1H). We selected the genes related to m^6^A modification and discovered that IGF2BP1 showed the most pronounced and consistent upregulation (Figure 1I). Immunohistochemistry revealed a progressive increase in IGF2BP1 expression across sequential PDX generations that developed resistance to gemcitabine (Figure 1J). Collectively, these findings establish IGF2BP1 as a key determinant of both acquired and intrinsic gemcitabine resistance in PDAC.

**FIGURE 1:**
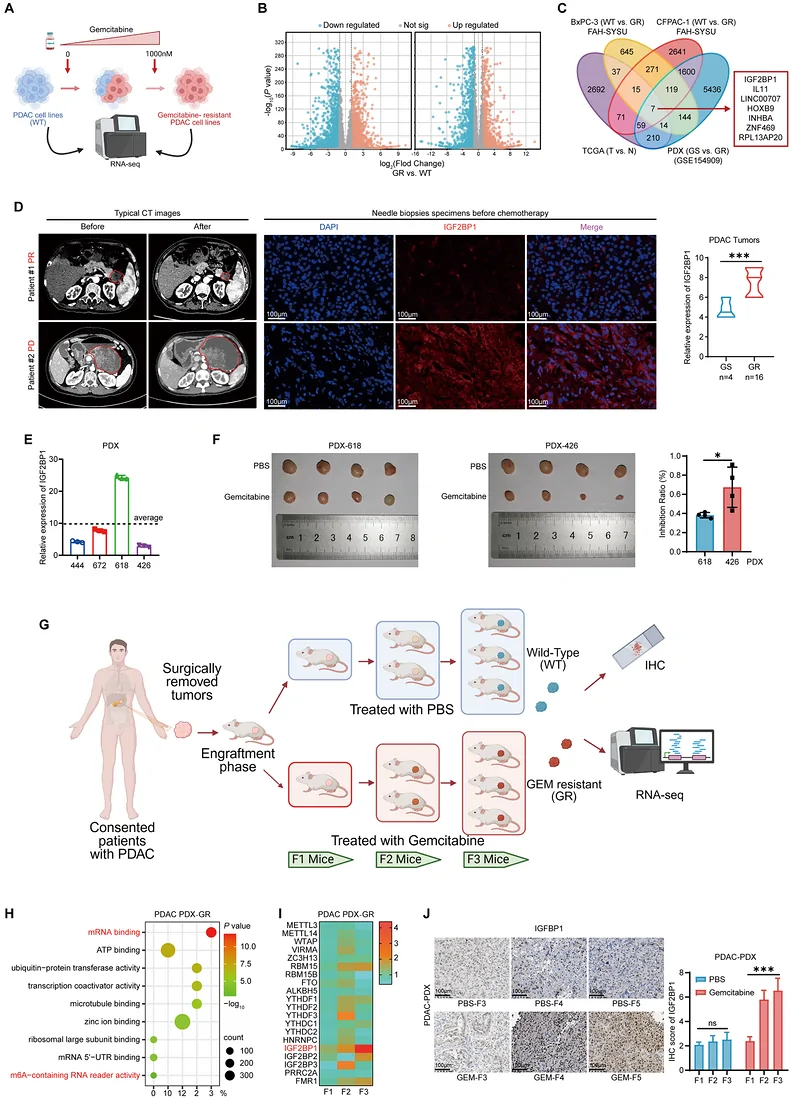
IGF2BP1 is highly expressed in gemcitabine‐resistant PDAC. (A) Schematic representation of the establishment and sequencing of gemcitabine‐resistant PDAC cells. Created with BioRender.com. (B) The volcano map shows the differentially expressed genes of GR PDAC cells compared with WT cells. (C) Venn diagram depicting upregulated genes in GR BxPC‐3 and CFPAC‐1 cells (sequencing data), gemcitabine‐resistant PDXs (data from GSE154909), and PDAC tumor tissues (data from TCGA database). (D) Representative CT images illustrating GEM‐sensitive (partial response, PR) and GEM‐resistant (progressive disease, PD) PDAC tumors before and after GEM‐based chemotherapy (left). Multi‐Immunofluorescence (mIF) staining for IGF2BP1(red) and DAPI (blue) in corresponding needle biopsy specimens obtained before chemotherapy (middle). Scale Bars, 100 µm. Quantification of IGF2BP1 expression based on mIF analysis in PD (*n* = 16) and PR (*n* = 4) patient samples (right), two‐tailed Student's t‐test for significance. (E) The expression of IGF2BP1 in establishment PDXs; (F) Gemcitabine has a poorer inhibitory effect on PDX with high expression of IGF2BP1 (*n* = 4 per group), two‐tailed Student's *t*‐test for significance; (G) Schematic representation of the establishment of wild‐type (WT) and gemcitabine‐resistant (GR) PDX models from PDAC; the constructed PDX tumors underwent IHC staining and sequencing. Created with BioRender.com. (H) Gene Ontology (GO) enrichment analysis of genes with progressively upregulated expression during the process of resistance formation; (I) The expression of genes related to m^6^A modification during the process of resistance formation; (J) Representative images of IHC staining for IGF2BP1 in different passages of WT and GR PDXs (*n* = 5 mice per group). Scale Bars, 100 µm (left). Quantification of IGF2BP1 expression based on IHC staining across different passages (right). Statistical significance was assessed using one‐way ANOVA followed by Tukey's post hoc test. Statistical significance for panels D and F was assessed using two‐tailed Student's t‐test. Data are presented as mean ± standard deviation (SD). ^*^ *p*< 0.05; ^**^ *p*< 0.01; ^***^ *p*< 0.001.

### 3.2 IGF2BP1 IS Associated With Gemcitabine Resistance in PDAC Both In Vitro and In Vivo

To functionally validate the role of IGF2BP1 in gemcitabine resistance, we modulated its expression in gemcitabine‐resistant PDAC cells (Figure 2A,B). Ectopic overexpression of IGF2BP1 elevated the IC_50_ for gemcitabine, enhanced cell proliferation, and promoted colony formation under gemcitabine treatment. Conversely, knockdown of IGF2BP1 significantly resensitized resistant cells to gemcitabine, manifesting as reduced IC_50_, suppressed proliferation, and decreased clonogenic survival (Figure 2C–E and Figure S2A–C). These in vitro findings were substantiated in vivo using xenograft models derived from the above engineered cells. Tumors with IGF2BP1 knockdown showed markedly slower growth under gemcitabine treatment, whereas IGF2BP1‐overexpressing xenografts exhibited accelerated tumor progression (Figure 2F and Figure S2D). The immunohistochemical results of Ki67 showed a consistent conclusion (Figure 2G and Figure S2E). Collectively, these results provide preliminary evidence that IGF2BP1 is a pivotal functional mediator of gemcitabine resistance in PDAC.

**FIGURE 2:**
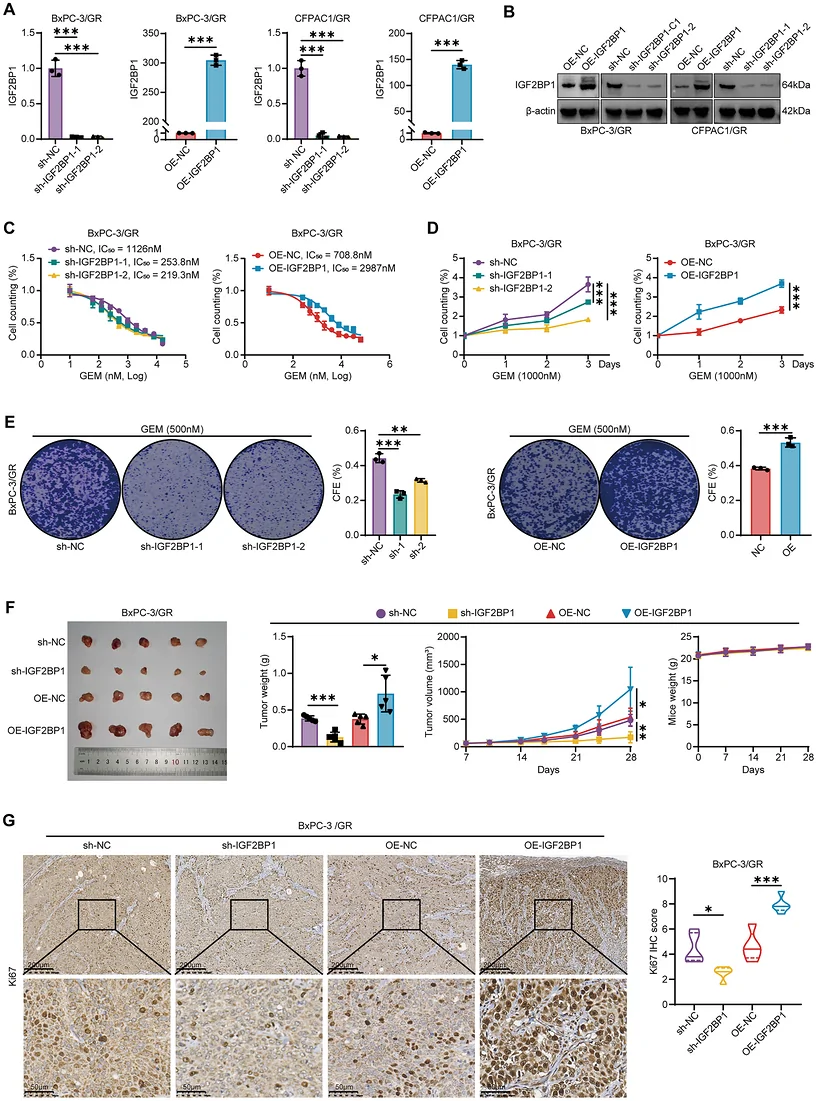
IGF2BP1 is associated with gemcitabine resistance in PDAC both in vitro and in vivo. (A) qPCR verification of IGF2BP1 knockdown and overexpression efficacy in BxPC‐3/GR and CFPAC‐1/GR cells. (B) Western blotting verification of IGF2BP1 knockdown and overexpression efficacy in BxPC‐3/GR and CFPAC‐1/GR cells; (C) Influence of IGF2BP1 knockdown and overexpression on IC_50_ values of gemcitabine in BxPC‐3/GR cells; (D) Effects of IGF2BP1 knockdown and overexpression on cell growth rate in BxPC‐3/GR cells treated with 1000 nm gemcitabine. (E) Effects of IGF2BP1 knockdown and overexpression on colony formation abilities in BxPC‐3/GR cells treated with 500 nm gemcitabine; representative images (left) and quantified outputs (right). (F) Mice harboring xenografts derived from IGF2BP1 knockdown and overexpressing BxPC‐3/GR cells were treated with gemcitabine (25 mg/kg, twice a week, intraperitoneally, *n* = 5 per group). Images of dissected tumors (left). Tumor weights (one‐way ANOVA followed by Tukey's post hoc test), tumor growth curves (repeated‐measures ANOVA), and mouse weights across groups (right); (G) Representative images of IHC staining for Ki67 in xenografts from each group (*n* = 5). Scale Bars, 200 µm (top) and 50 µm (bottom). Quantification of Ki67‐positive cells was analyzed using one‐way ANOVA followed by Tukey's post hoc test. Statistical significance for panels A–E was assessed using two‐tailed Student's t‐test or one‐way ANOVA followed by Dunnett's post hoc test, as appropriate. Data are presented as mean ± standard deviation (SD). ^*^ *p*< 0.05; ^**^ *p*< 0.01; ^***^ *p*< 0.001.

### 3.3 IGF2BP1 Stabilizes FTH1 mRNA in an M^6^A‐Dependent Manner

To investigate the mechanism by which IGF2BP1 regulates gemcitabine resistance in PDAC, we performed an integrated multi‐omics analysis. MeRIP‐seq identified m^6^A‐modified transcripts, and RIP‐seq using an IGF2BP1‐specific antibody revealed its direct binding targets. Transcriptome sequencing of IGF2BP1‐knockdown cells treated with gemcitabine revealed genes regulated by IGF2BP1(Figure 3A). Integrative Venn analysis of these three datasets identified FTH1 as a key candidate target that is m^6^A‐modified, directly bound by IGF2BP1, and post‐transcriptionally regulated by IGF2BP1(Figure 3B). Integrative genomics viewer (IGV) plots visualizing m^6^A peaks and IGF2BP1 binding sites on FTH1 mRNAs in gemcitabine‐resistant PDAC cells (Figure 3C). RIP‐qPCR and methylated RNA immunoprecipitation (meRIP‐qPCR) assays confirmed the direct binding of IGF2BP1 to FTH1 mRNA (Figure 3D and Figure S3A) and the presence of m^6^A modifications on FTH1 transcripts (Figure 3E). Consistently, modulation of IGF2BP1 expression resulted in corresponding changes in FTH1 mRNA levels (Figure 3F and Figure S3B). Actinomycin D chase assays demonstrated that IGF2BP1 significantly prolonged the half‐life of FTH1 mRNA, indicating enhanced transcript stability (Figure 3G and Figure S3C).

**FIGURE 3:**
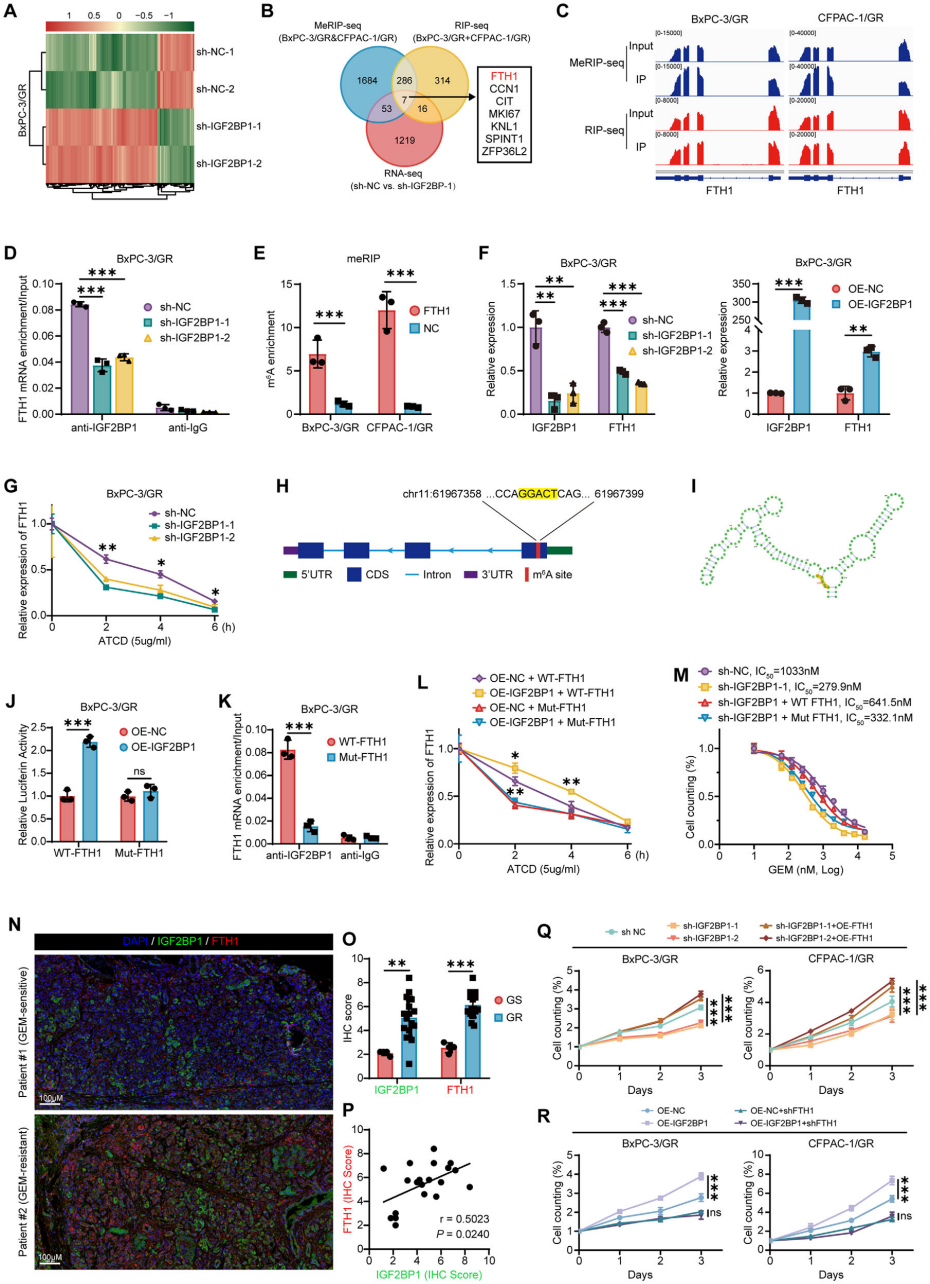
IGF2BP1‐Mediated m^6^A Modification Sustains FTH1 mRNA Expression. (A) Heatmap showing transcriptomic alterations following IGF2BP1 knockdown in BxPC‐3/GR cells; (B) Venn diagram illustrating the overlap among genes harboring m^6^A modifications, IGF2BP1 binding sites, and genes significantly downregulated upon IGF2BP1 knockdown in PDAC cells; (C) Integrative genomics viewer (IGV) plots visualizing m^6^A peaks and IGF2BP1 binding sites on FTH1 mRNAs in gemcitabine‐resistant PDAC cells; (D) RIP‐qPCR analysis using an anti‐IGF2BP1 antibody or IgG control demonstrating the direct interaction between IGF2BP1 and FTH1 mRNA in BxPC‐3/GR cells. (in vivo) Methylated RNA immunoprecipitation (meRIP‐qPCR) using an anti‐m^6^A antibody confirming m^6^A modifications on FTH1 mRNA. (F) Quantitative analysis of FTH1 mRNA expression levels following IGF2BP1 knockdown or overexpression in BxPC‐3/GR cells. (G) IGF2BP1‐knockdown BxPC‐3/GR cells were treated with 5 µg/mL actinomycin D (ActD) to inhibit transcription, and the decay rate of FTH1 mRNAs was measured by qPCR. (H) Schematic illustration of the m^6^A mutation sites within the FTH1 transcript. (I) Prediction of very high‐confidence m^6^A modification sites in FTH1 by SRAMP. (J) Dual‐luciferase reporter assay showing that mutation of the FTH1 m^6^A site significantly impaired IGF2BP1‐mediated regulation of FTH1 in BxPC‐3/GR cells. (K) RIP‐qPCR analysis demonstrating that mutation of the FTH1 m^6^A site markedly reduced the binding affinity of IGF2BP1 to FTH1 transcripts in BxPC‐3/GR cells. (L) Actinomycin D (ActD) chase assay showing that mutation of the FTH1 m^6^A site significantly decreased FTH1 mRNA stability in BxPC‐3/GR cells. (M) IC_50_ analysis showing that mutation of the FTH1 m^6^A site significantly increased the sensitivity of PDAC cells to gemcitabine in BxPC‐3/GR cells; (N) Multiplex immunofluorescence (mIF) staining of IGF2BP1 (green), FTH1 (red), and nuclei (DAPI, blue) in gemcitabine‐resistant and gemcitabine‐sensitive PDAC needle biopsy samples obtained prior to chemotherapy (*n* = 20). Scale Bars, 100 µm. (O) Quantitative immunofluorescence scoring of IGF2BP1 and FTH1 expression in gemcitabine‐resistant vs. gemcitabine‐sensitive biopsy specimens (*n* = 20). (P) Correlation analysis of IGF2BP1 and FTH1 immunofluorescence scores across PDAC biopsy samples (*n* = 20); (Q) FTH1 overexpression rescues the proliferation defect caused by IGF2BP1 knockdown under 500 nm gemcitabine. (R) FTH1 knockdown rescues the excessive proliferation caused by IGF2BP1 overexpression under 500 nm gemcitabine. Statistical significance for panels D and G was determined using one‐way ANOVA followed by Dunnett's post hoc test. Statistical significance for panels E, J, K, and O was determined using a two‐tailed Student's *t*‐test. For panels F, either a two‐tailed Student's *t*‐test or one‐way ANOVA followed by Dunnett's post hoc test was applied, as appropriate. For panels L, Q, and R, statistical significance was determined using one‐way ANOVA followed by Tukey's post hoc test, as appropriate. Data are presented as mean ± standard deviation (SD). ^*^ *p*< 0.05; ^**^ *p*< 0.01; ^***^ *p*< 0.001.

To further validate the m^6^A dependency of this interaction, we generated site‐specific mutations at the predicted m^6^A site within the FTH1 transcript. The mutated adenosine was located within the major MeRIP‐seq peak and was identified as a very high‐confidence m^6^A site by SRAMP, with additional support from RMBase annotations (Figure 3H,I). Dual‐luciferase reporter assays, RIP‐qPCR, and Actinomycin D chase experiments showed that mutation of this m^6^A site markedly impaired IGF2BP1 binding and significantly reduced FTH1 mRNA stability (Figure 3J–L and Figure S3D–F), supporting a site‐specific m^6^A‐dependent regulatory mechanism. Functionally, mutation of the FTH1 m^6^A site significantly increased cellular sensitivity to gemcitabine, as reflected by reduced IC_50_ values (Figure 3M and Figure S3G), further demonstrating that m^6^A‐dependent stabilization of FTH1 contributes to IGF2BP1‐mediated chemoresistance.

To validate the clinical relevance of this axis, we collected 45 pretreatment biopsy samples from PDAC patients who subsequently received gemcitabine‐based combination chemotherapy and were followed longitudinally. Based on radiological response evaluation, 16 patients were classified as progressive disease (PD), indicating gemcitabine resistance, whereas 4 achieved partial response (PR), indicating gemcitabine sensitivity. The remaining 25 patients were classified as stable disease (SD) and were excluded to minimize phenotypic heterogeneity. Multiplex immunofluorescence analysis of the PD and PR cohorts revealed a significant positive correlation between IGF2BP1 and FTH1 expression, both of which were strongly associated with gemcitabine resistance (Figure 3N–P and Figure S3H).

To determine whether IGF2BP1‐mediated FTH1 upregulation contributes to gemcitabine resistance, we knocked down FTH1 in IGF2BP1‐overexpressing cells and conversely overexpressed FTH1 in IGF2BP1‐knockdown cells. Rescue experiments demonstrated that the effects of IGF2BP1 on cell proliferation and accumulation could be reversed by reciprocal modulation of FTH1, indicating that FTH1 is an essential downstream effector of IGF2BP1 (Figure 3Q,R). Together, these results indicate that IGF2BP1 recognizes and binds to m^6^A sites on FTH1 mRNA, enhancing its stability and preventing degradation, thereby promoting gemcitabine resistance.

### 3.4 Stress Granule Assembly by IGF2BP1/G3BP1 Stabilizes FTH1 mRNA

We next explored the mechanism by which IGF2BP1 stabilizes FTH1 mRNA. Co‐immunoprecipitation using Flag‐tagged IGF2BP1 followed by liquid chromatography‐tandem mass spectrometry (LC‐MS/MS) identified potential IGF2BP1‐binding partners (Figure 4A). Among these, the stress granule core protein Ras‐GTPase‐activating protein (GAP)‐binding protein 1 (G3BP1) emerged as a high‐confidence interactor based on peptide abundance and sequence coverage (Figure 4B,C). A schematic representation of the predicted binding conformations between IGF2BP1 and G3BP1 was derived from protein‐protein interaction modeling (Figure 4D). This interaction was confirmed through endogenous co‐IP assays (Figure 4E). Functionally, knockdown of G3BP1 abolished the alterations in FTH1 expression levels induced by IGF2BP1 modulation (Figure 4F and Figure S4), indicating that G3BP1 is essential for the regulation of FTH1 by IGF2BP1. Consistently, either genetic silencing of G3BP1 or pharmacological inhibition of stress granule (SG) formation using FAZ‐3532 significantly reduced FTH1 mRNA stability, further supporting the protective role of SGs in maintaining FTH1 transcripts (Figure 4G).

**FIGURE 4:**
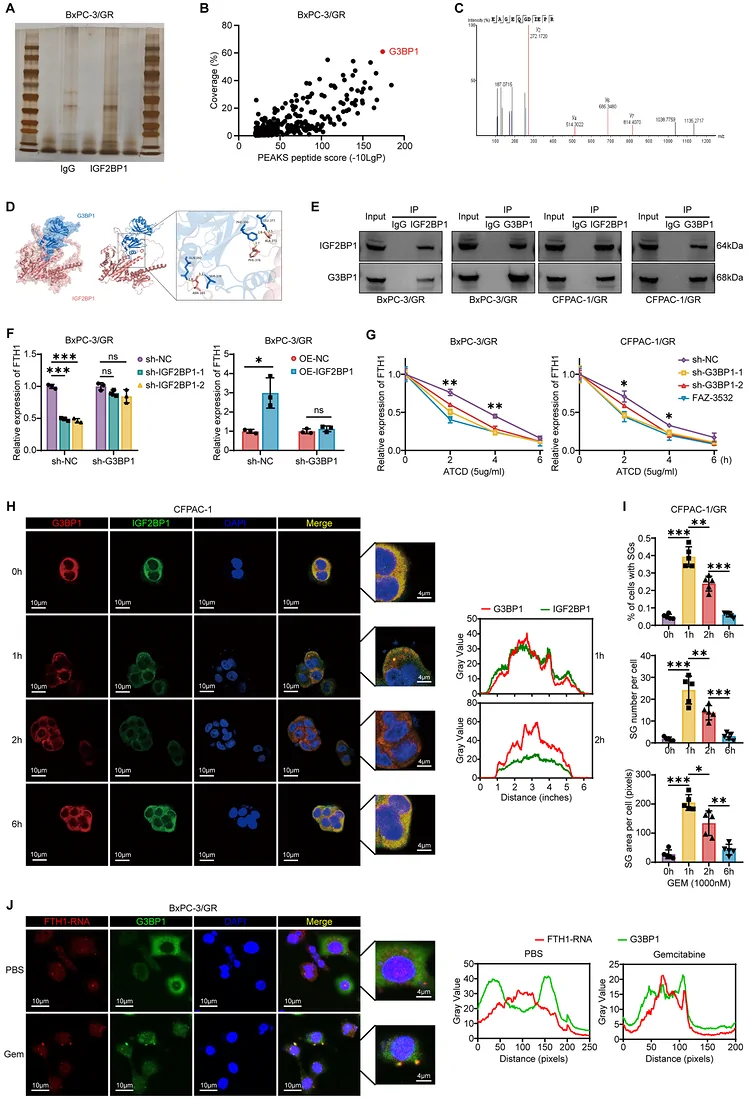
Stress Granule Formation by G3BP1 and IGF2BP1 Inhibits FTH1 mRNA Degradation. (A) Silver staining identified the proteins bound by IGF2BP1; (B) IGF2BP1‐bound proteins in BxPC‐3/GR cells were purified by Co‐IP and identified by LC‐MS/MS. Scatterplots display proteins identified by LC‐MS/MS; (C) Peptide‐spectrum match (PSM) of G3BP1 identified by LC‐MS/MS, indicating a high‐confidence interaction with IGF2BP1; (D) Schematic representation of the predicted binding conformations between IGF2BP1 and G3BP1, based on protein‐protein interaction modeling; (E) Co‐IP assays confirming the physical interaction between IGF2BP1 and G3BP1 in gemcitabine‐resistant PDAC cells; (F) qPCR analysis showing that knockdown of G3BP1 reverses the effects of IGF2BP1 depletion or overexpression on FTH1 mRNA levels in BxPC‐3/GR cells, two‐tailed Student's t‐test or one‐way ANOVA with Dunnett's test for significance. (G) Actinomycin D (ActD) chase assay showing that knockdown of G3BP1 or pharmacological inhibition of SG formation using FAZ‐3532 significantly reduced FTH1 mRNA stability. (H) Immunofluorescence staining revealed that gemcitabine treatment promotes the colocalization of IGF2BP1 and G3BP1 in stress granules. Scale Bars, 10 µm (left) and 4 µm (right). Time‐course immunofluorescence colocalization analysis at specific time points (1 and 2 h) confirmed that IGF2BP1 and G3BP1 were early recruited to newly formed SGs, exhibiting strong colocalization, and subsequently underwent gradual disassembly (right). (I) Quantification of SG‐positive cells, SG number per cell, and SG area per cell at different time points following gemcitabine treatment (*n* = 5). (J) RNA FISH combined with immunofluorescence showing the recruitment of FTH1 mRNA into G3BP1‐positive newly formed SGs and their strong colocalization in gemcitabine‐treated PDAC cells (left), with quantitative colocalization analysis shown on the right. Statistical significance for panels G and I was assessed using one‐way ANOVA with Tukey's post hoc test, as appropriate. Data are presented as mean ± standard deviation (SD). ^*^ *p*< 0.05; ^**^ *p*< 0.01; ^***^ *p*< 0.001.

Immunofluorescence analysis revealed that both IGF2BP1 and G3BP1 exhibited diffuse cytosolic localization in CFPAC‐1/GR cells. Under gemcitabine treatment, both IGF2BP1 and G3BP1 were recruited to newly formed stress granules (SG) and exhibited strong colocalization, suggesting cooperative involvement in SG assembly. Within 2 h of gemcitabine treatment, SGs underwent fragmentation. Concomitantly, IGF2BP1 dissociated from SGs and redistributed diffusely throughout the cytosol—suggestive of translational reactivation—whereas G3BP1 persisted in SG remnants. Complete SG disassembly occurred by 6 h post‐recovery, coinciding with G3BP1 reversion to homogeneous cytoplasmic distribution (Figure 4H,I). To directly determine whether FTH1 mRNA is recruited into SGs, we performed RNA‐FISH combined with immunofluorescence staining for G3BP1. The results showed robust colocalization of FTH1 mRNA with G3BP1‐positive SGs following gemcitabine treatment (Figure 4J). Collectively, these data implicate G3BP1‐IGF2BP1‐coordinated stress granule formation in shielding FTH1 mRNA from degradation.

### 3.5 The IGF2BP1‐FTH1 Axis Regulates Iron Homeostasis and Inhibits Ferroptosis

To determine whether ferroptosis contributes to gemcitabine‐induced cell death in PDAC, we first examined ferroptotic features in gemcitabine‐treated cells. Transmission electron microscopy revealed that gemcitabine‐treated PDAC cells exhibited typical morphological features of ferroptosis, including mitochondrial shrinkage, reduced cristae, and increased membrane density (Figure 5A). Consistently, gemcitabine treatment significantly elevated intracellular ROS accumulation, indicating enhanced oxidative stress (Figure S5A). Given the central role of FTH1 in iron sequestration and ferroptosis regulation, we next investigated whether IGF2BP1‐mediated upregulation of FTH1 contributes to gemcitabine resistance through ferroptosis suppression. To this end, we knocked down FTH1 in IGF2BP1‐overexpressing cells and, conversely, overexpressed FTH1 in IGF2BP1‐depleted cells. Rescue experiments demonstrated that IGF2BP1‐induced changes in lipid peroxidation (LPO), a hallmark of ferroptosis, were reversed by reciprocal modulation of FTH1 (Figure 5B and Figure S5B). To further determine whether this effect depends on m^6^A modification of FTH1, we introduced site‐specific mutations at the FTH1 m^6^A site and assessed ferroptotic activity. Compared with the wild‐type FTH1 rescue group, cells expressing m^6^A‐mutant FTH1 exhibited significantly elevated LPO levels (Figure S5C), indicating that m^6^A‐dependent stabilization of FTH1 is required for IGF2BP1‐mediated ferroptosis suppression.

**FIGURE 5:**
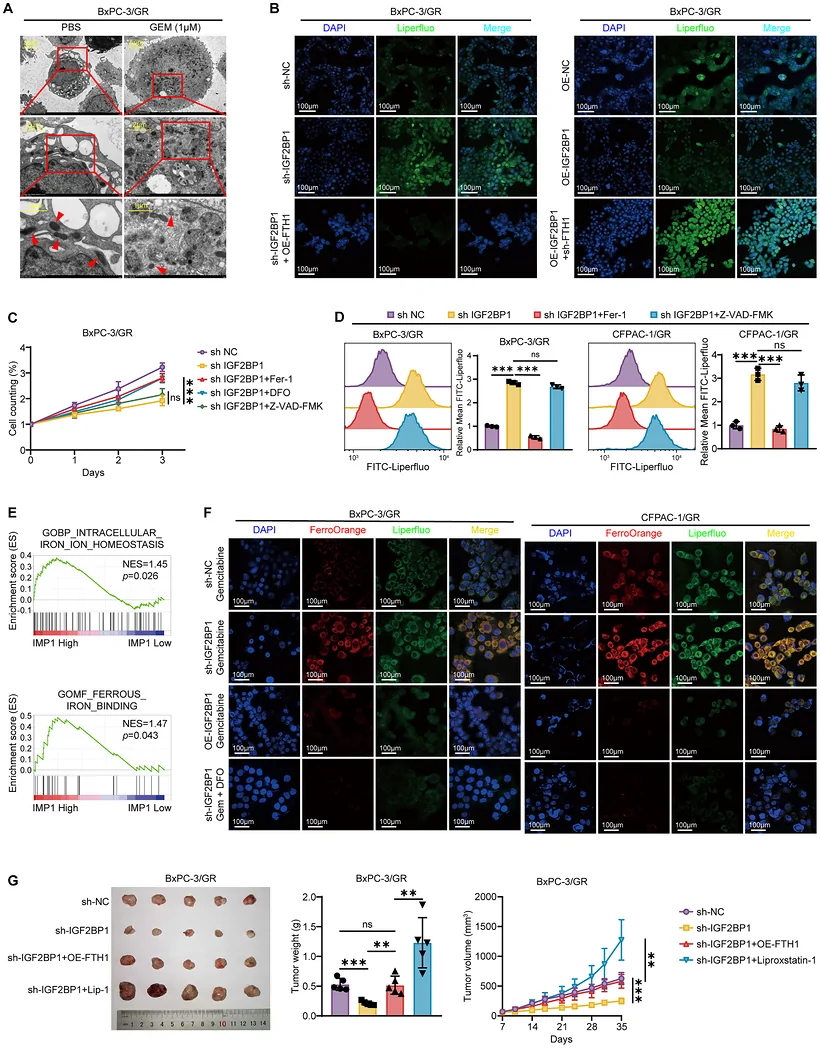
The IGF2BP1‐FTH1 Axis Regulates Iron Homeostasis and Inhibits Ferroptosis. (A) Transmission electron microscopy shows mitochondrial shrinkage and cristae loss in gemcitabine‐treated PDAC cells (red arrows). Scale Bars, 5 µm (left), 2 µm (middle), and 1 µm (right); (B) Quantification of lipid peroxidation (LPO) levels showing that IGF2BP1 knockdown increased, whereas IGF2BP1 overexpression decreased LPO levels under gemcitabine treatment; these effects were reversed by reciprocal modulation of FTH1, Scale Bars, 100 µm (*n* = 5). (C) Cell viability analysis of BxPC‐3/GR cells treated with gemcitabine in combination with IGF2BP1 knockdown and ferroptosis inhibitors (Ferrostatin‐1 and deferoxamine) or apoptosis inhibitor Z‐VAD‐FMK. (D) Quantification of lipid peroxidation (LPO) levels in BxPC‐3/GR cells following IGF2BP1 knockdown in the presence of the ferroptosis inhibitors Ferrostatin‐1 (Fer‐1) and deferoxamine (DFO), or Z‐VAD‐FMK. (E) GO enrichment analysis reveals enrichment of IGF2BP1‐associated genes in *intracellular iron ion homeostasis* and *ferrous ion binding* pathways in PDAC, (F) Fluorescent probes (FerroOrange and Liperfluo) show that IGF2BP1 knockdown elevates, and overexpression reduces, intracellular Fe^2^^+^ and LPO levels. Scale Bars, 100 µm (*n* = 5). (G) Xenograft models established using IGF2BP1‐knockdown BxPC‐3/GR cells, with ectopic FTH1 overexpression or treatment with the ferroptosis inhibitor Liproxstatin‐1 (Lip‐1), followed by gemcitabine treatment (25 mg/kg, twice a week, intraperitoneally, *n* = 5 per group). Images of dissected tumors (left). Tumor weights (one‐way ANOVA with Tukey's post hoc test) and tumor growth (repeated‐measures ANOVA) curves across groups (right). Statistical significance for panels C–D was assessed using one‐way ANOVA with Tukey's post hoc test, as appropriate. Data are presented as mean ± standard deviation (SD). ^*^ *p*< 0.05; ^**^ *p*< 0.01; ^***^ *p*< 0.001.

To further validate ferroptosis specificity, IGF2BP1‐silenced cells were treated with the canonical ferroptosis inhibitors Ferrostatin‐1 (Fer‐1) and deferoxamine (DFO), as well as the apoptosis inhibitor Z‐VAD‐FMK. Both Fer‐1 and DFO significantly restored cell viability and reduced LPO accumulation induced by IGF2BP1 depletion, whereas Z‐VAD‐FMK showed no significant protective effect (Figure 5C,D and Figure S5D), supporting that ferroptosis is a major downstream consequence of IGF2BP1 depletion under gemcitabine stress.

To further elucidate how IGF2BP1 regulates ferroptosis, Gene Set Enrichment Analysis (GSEA) of TCGA PDAC datasets revealed that high IGF2BP1 expression was significantly associated with pathways related to *Intracellular Iron Ion Homeostasis* and *Ferrous Iron Binding* in PDAC (Figure 5E). Consistent with these findings, IGF2BP1 knockdown increased intracellular Fe^2^^+^ and LPO levels, whereas IGF2BP1 overexpression reduced both; these effects were abrogated by treatment with the iron chelator deferoxamine (DFO) (Figure 5F and Figure S5E).

To validate the functional significance of the IGF2BP1‐FTH1 axis in vivo, we established xenograft models and performed rescue experiments under gemcitabine treatment. Tumors with IGF2BP1 knockdown exhibited significantly reduced growth compared with control tumors, indicating enhanced gemcitabine sensitivity. Notably, ectopic expression of FTH1 or treatment with the ferroptosis inhibitor Liproxstatin‐1 (Lip‐1) largely restored tumor growth (Figure 5G), suggesting that the tumor growth inhibition induced by IGF2BP1 depletion is mediated, at least in part, through ferroptosis. Consistently, immunohistochemical analysis showed increased 4‐HNE accumulation and reduced GPX4 expression in IGF2BP1‐silenced tumors, indicating enhanced ferroptotic activity. In contrast, FTH1 overexpression or Lip‐1 treatment significantly attenuated these changes, as evidenced by reduced 4‐HNE levels and restored GPX4 expression (Figure S5F).

Collectively, these data indicate that IGF2BP1 stabilizes FTH1 mRNA to preserve iron homeostasis, thereby limiting oxidative stress and lipid peroxidation, suppressing gemcitabine‐induced ferroptosis, and ultimately promoting chemoresistance in PDAC.

### 3.6 Targeting IGF2BP1 Sensitizes PDAC to Gemcitabine

Considering the crucial role of the IGF2BP1‐FTH1 axis in regulating ferroptosis and gemcitabine resistance, we hypothesized that targeting IGF2BP1 could restore gemcitabine sensitivity in PDAC. To test this, we evaluated the therapeutic potential of BTYNB, the most recently developed inhibitor of IGF2BP1. In vitro, BTYNB markedly inhibited the survival of gemcitabine‐resistant PDAC cells at low concentrations (Figure 6A) and exhibited strong synergistic effects with gemcitabine (combination index CI < 1) (Figure 6B,C and Figure S6A,B). Cell viability assays and colony formation confirmed the synergistic effect of the combined treatment, which resulted in a more potent inhibition of proliferation than either single agent (Figure 6D,E and Figure S6C,D). Mechanistically, BTYNB treatment significantly reduced FTH1 expression, suggesting that inhibition of IGF2BP1 disrupts the IGF2BP1‐FTH1 regulatory axis (Figure 6F and Figure S6E). To further determine whether FTH1 mediates the sensitizing effect of BTYNB, we performed rescue experiments by ectopically overexpressing FTH1. Notably, FTH1 restoration partially reversed the enhanced sensitivity to gemcitabine induced by BTYNB, as evidenced by increased IC_50_ values compared with the BTYNB combination group (Figure 6G). These findings indicate that the therapeutic effect of BTYNB is mediated, at least in part, through suppression of the IGF2BP1‐FTH1 axis.

**FIGURE 6:**
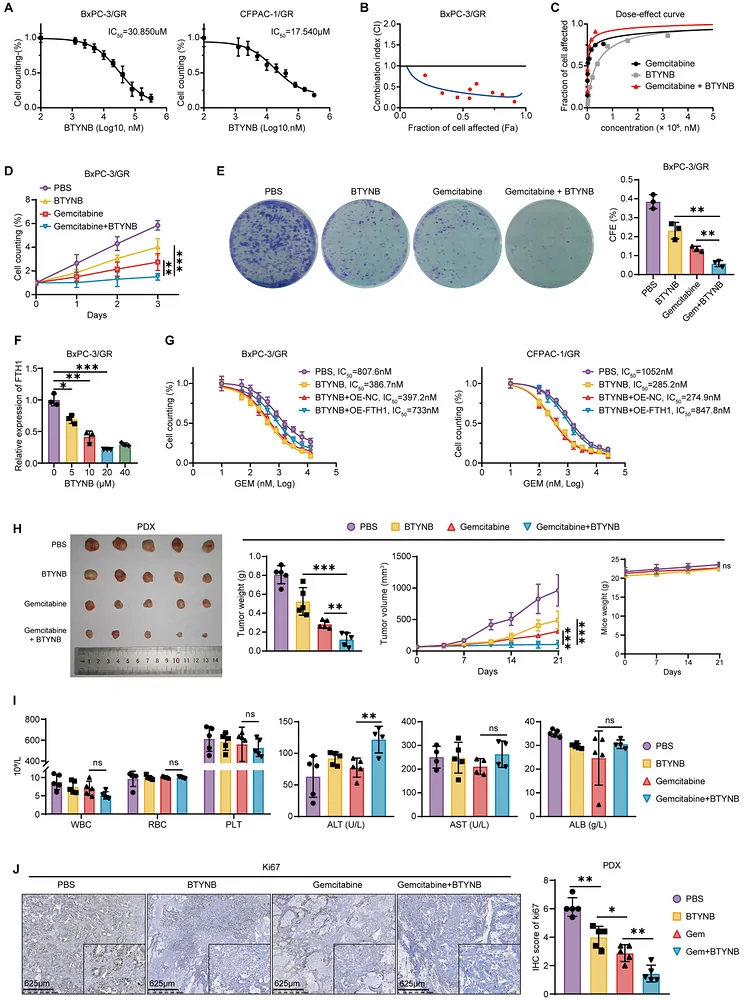
Targeting IGF2BP1 sensitizes PDAC to Gemcitabine (A) IC_50_ values of BTYNB in gemcitabine‐resistant (GR) PDAC cells; (B) Combination Index (CI) depicting the synergistic effect of gemcitabine and BTYNB on BxPC‐3/GR cells; (C) Dose‐response curves illustrating the individual and combined treatment effects of gemcitabine and BTYNB on BxPC‐3/GR cells; (D) Cell growth curves of BxPC‐3/GR cells treated with PBS, BTYNB, gemcitabine, or the combination of gemcitabine and BTYNB. (E) Colony formation abilities revealing the effects of PBS, BTYNB, gemcitabine, or the combination of gemcitabine and BTYNB on BxPC‐3/GR cells (*n* = 5). (F) qPCR analysis showing that BTYNB treatment significantly reduced FTH1 mRNA expression in BxPC‐3/GR cells. (G) IC_50_ analysis demonstrating that BTYNB significantly enhanced gemcitabine sensitivity in PDAC cells, while ectopic overexpression of FTH1 partially rescued this effect. (H) Mice harboring gemcitabine‐resistant PDXs were treated with PBS, BTYNB, gemcitabine, or the combination of gemcitabine and BTYNB. Tumor photos, tumor weights (one‐way ANOVA with Tukey's post hoc test), tumor volume growth curves (repeated‐measures ANOVA), and mouse weights across groups (*n* = 5 per group) are displayed. (I) Statistical analysis showing blood routine examination and liver function of mice in different treatment groups (*n* = 5). (J) Typical images of IHC staining for Ki67 in gemcitabine‐resistant PDXs from different treatment groups (*n* = 5 per group). Scale Bars, 625 µm (left). The IHC score of Ki67 in gemcitabine‐resistant PDXs from different treatment groups (right). Statistical significance for panels D‐F and I‐J was assessed using one‐way ANOVA with Tukey's post hoc test, as appropriate. Data are presented as mean ± standard deviation (SD). ^*^ *p*< 0.05; ^**^ *p*< 0.01; ^***^ *p*< 0.001.

In vivo, gemcitabine‐resistant PDX models treated with the combination of BTYNB and gemcitabine developed the smallest tumors among all treatment groups (Figure 6H). Importantly, this enhanced antitumor efficacy was not accompanied by increased systemic toxicity, as evidenced by unchanged body weight, blood parameters, and liver/kidney function compared to gemcitabine monotherapy (Figure 6I and Figure S6F). Further corroborating the treatment efficacy, tumors from the combination group showed the lowest expression of the proliferation marker Ki67 (Figure 6J). Immunohistochemical analysis confirmed that the IGF2BP1 inhibitor BTYNB markedly reduced FTH1 expression levels (Figure S6G). In conclusion, pharmacological inhibition of IGF2BP1 by BTYNB effectively sensitizes gemcitabine‐resistant PDAC to chemotherapy, supporting a preclinical rationale for IGF2BP1‐targeted combination strategies to overcome gemcitabine resistance.

## 4 Discussion

PDAC remains one of the most lethal malignancies, largely due to its low resection rate and high recurrence, which make chemotherapy indispensable for most patients [18]. Despite the introduction of combination regimens, gemcitabine‐based chemotherapy remains a cornerstone of treatment for a substantial proportion of patients [19]. However, its clinical efficacy is severely constrained by the dynamic evolution of chemoresistance.

N6‐methyladenosine (m^6^A) has emerged as a pivotal epitranscriptomic regulator of cancer progression and therapeutic response, with increasing evidence demonstrating its critical role in shaping gemcitabine resistance in PDAC through diverse mechanisms, including PTEN signaling [20], cell‐cycle quiescence [21], translational control [22], and metabolic reprogramming [23]. Against this mechanistic backdrop, our study identified that the m^6^A reader IGF2BP1 exhibited the strongest and most consistent upregulation across gemcitabine‐resistant models, and its elevated expression in pretreatment biopsies was significantly associated with gemcitabine resistance, prompting a systematic investigation of its downstream effector mechanisms.

IGF2BP1 has been implicated in chemoresistance across multiple malignancies​ [24, 25]. In PDAC, IGF2BP1 promotes chemoresistance through several m^6^A‐dependent mechanisms: stabilizing SH3BP5‑AS1 [26]; interacting with RRP9 to activate AKT signaling [27], and maintaining an EP300‐CMTM6‐IGF2BP1 feedback loop via m^6^A‐modified EP300 and MYC transcripts, thereby enhancing tumor stemness and therapeutic resistance [28]. Among IGF2BP1's target transcripts, we identified FTH1 as a direct and functionally critical m^6^A‐modified substrate. By integrating transcriptomic profiling, m^6^A‐RNA immunoprecipitation sequencing, and IGF2BP1‐RIP sequencing, we demonstrate that IGF2BP1 directly binds to m^6^A‐modified FTH1 mRNA, thereby enhancing its transcript stability. This finding extends the known oncogenic repertoire of IGF2BP1 in PDAC‐previously linked to AKT signaling and c‐Myc stabilization‐toward a previously uncharacterized role in iron homeostasis, identifying IGF2BP1‐FTH1 as a novel m^6^A reader‐target axis in this disease.

A central mechanistic finding of this study concerns stress granules (SGs)‐dynamic, membrane‐less cytoplasmic condensates that assemble in response to cellular stress and sequester mRNAs and RNA‐binding proteins, thereby modulating mRNA stability, translation, and fate. Increasing evidence implicates SG dynamics in tumor stress adaptation and chemoresistance across multiple cancer types [29], yet their role in PDAC chemoresistance remains largely unexplored [30]. Here, we show that IGF2BP1 protects FTH1 mRNA not merely through direct binding, but through SG‐mediated transcript sequestration. The identification of G3BP1‐the master nucleator of SG assembly‐as an IGF2BP1‐interacting partner reveals that, under gemcitabine‐induced stress, IGF2BP1 co‐opts SG formation to shield FTH1 mRNA from degradation, releasing it for translation once the stress subsides. Quantitative analysis of SG dynamics further supports their functional role in RNA protection under chemotherapeutic stress. This represents a previously unappreciated layer of post‐transcriptional regulation in which an m^6^A reader exploits SG dynamics to determine the fate of a specific resistance‐associated transcript, adding chemoresistance to the growing list of cancer hallmarks governed by SG biology in PDAC. It should be noted that the precise molecular determinants of the IGF2BP1‐G3BP1 interaction, and the mechanism by which FTH1 mRNA is selectively recruited into SGs, remain to be fully elucidated.

Functionally, this IGF2BP1‐SG‐TH1 axis converges on ferroptosis suppression through the regulation of iron homeostasis. Ferritin, composed of FTH1 and FTL subunits, serves as the principal intracellular iron storage complex; its heavy chain, FTH1, possesses ferroxidase activity that oxidizes labile Fe^2^^+^ to Fe^3^^+^ for safe sequestration [16]. Loss of this ferroxidase activity permits accumulation of redox‐active Fe^2^^+^, which catalyzes Fenton chemistry and drives the iron‐dependent lipid peroxidation that defines ferroptosis [31]. By sustaining FTH1 levels under gemcitabine‐induced stress, IGF2BP1 effectively dampens this vulnerability, allowing resistant cells to tolerate chemotherapy‐induced oxidative and iron stress. Our in vivo rescue experiments support a causal role for this axis in tumor growth under IGF2BP1 perturbation, mediated specifically through ferroptosis. Together, these findings position the IGF2BP1‐SG‐FTH1 axis as a core ferroptosis‐suppressive mechanism sustaining gemcitabine resistance in PDAC.

From a clinical perspective, IGF2BP1 holds dual promise: its expression in pretreatment biopsies correlates with gemcitabine resistance and may serve as a predictive biomarker to guide patient stratification, while its pharmacological inhibition by BTYNB [32, 33]‐which carries a favorable therapeutic window given IGF2BP1's near‐absent expression in normal adult tissues​ [34]‐offers a rational strategy to resensitize resistant tumors to gemcitabine. Prospective validation of these translational implications in larger cohorts will be an important next step toward clinical implementation.

Despite these findings, several limitations should be acknowledged. First, although our data support a central role for the IGF2BP1‐FTH1 axis in ferroptosis regulation, additional downstream effectors of IGF2BP1 may also contribute to chemoresistance. In addition, while our results strongly support ferroptosis as a key downstream pathway, the potential involvement of other regulated cell death programs cannot be fully excluded. Second, while we demonstrate that IGF2BP1 facilitates stress granule‐mediated protection of FTH1 transcripts, the precise structural basis underlying IGF2BP1‐G3BP1 interaction remains to be further defined. Finally, although BTYNB showed promising synergistic efficacy with gemcitabine in preclinical models, its pharmacokinetic properties and safety profile require further validation before clinical translation.

## 5 Conclusion

This study identifies IGF2BP1 as a central regulator of gemcitabine resistance in PDAC, uncovering a previously unrecognized link between m^6^A‐dependent RNA stabilization, stress granule dynamics, and ferroptosis suppression. The IGF2BP1‐FTH1 axis emerges as a key mechanism by which PDAC cells exploit post‐transcriptional regulation to preserve iron homeostasis and evade ferroptosis under therapeutic stress, offering new insight into how epitranscriptomic reprogramming shapes chemoresistance. Importantly, these findings position IGF2BP1 as both a predictive biomarker for patient stratification and a therapeutically actionable target. Combining IGF2BP1 inhibition with gemcitabine‐based chemotherapy may therefore represent a promising strategy to overcome resistance and improve clinical outcomes in PDAC.

## Author Contributions

X. Yin and Y. Huang conceived the project and designed the experiments. Y. Zhu, J. Xie, and J. Chen collected all the tissue samples. J. Ye, D. Fang, and Z. Zhao analyzed all the clinical information. S. Lu, Z. Liu, and M. Ma provided useful suggestions for this manuscript. Y, Zhu and Y, Huang wrote the manuscript with input from all authors.

## Ethics Statement

All experiments involving human subjects were conducted in accordance with the Code of Ethics of the World Medical Association (Declaration of Helsinki). All procedures complied with relevant laws and institutional guidelines. Ethics approval for the use of human tumor specimens was granted by the Internal Review Board and Ethics Committee of FAHSYSU (IIT‐2021‐719). Written informed consent was obtained from all patients prior to sample collection.

All animal experiments were approved by the Institutional Review Board of FAHSYSU (2025‐053). The animals received humane care according to the guidelines outlined in the “Guide for the Care and Use of Laboratory Animals”.

Written informed consent was obtained from all patients before sample collection.

## Conflicts of Interest

The authors declare no conflicts of interest.

## Supporting information




**Supporting File**: advs77048‐sup‐0001‐SuppMat.docx.

## Data Availability

The MeRIP‐seq, RIP‐seq, and RNA‐seq data generated in this study have been deposited in the Gene Expression Omnibus (GEO) under accession numbers GSE337101, GSE337103, and GSE337104.
