# Supplementary material for: Ferroptosis Suppression by the M6A Reader IGF2BP1 Underlies Gemcitabine Resistance in Pancreatic Ductal Adenocarcinoma
Source: Adv Sci (Weinh). 2026 Aug 10:e77048. Online ahead of print. doi: 10.1002/advs.77048 (PMC13455834; doi:10.1002/advs.77048)
Supplement: Supplementary file 1 [file ADVS-9999-e77048-s001.docx]

Supplementary information for

**Ferroptosis Suppression by the m^6^A Reader IGF2BP1 Underlies Gemcitabine Resistance in Pancreatic Ductal Adenocarcinoma**

**Authors:** Ying-Qin Zhu^1†^; Yue Huang^2†^; Jing-Yuan Ye^1,3†^; Si-Yuan Lu^1†^; Zhi-De Liu^1^; Ming-Jian Ma^1^; De-Liang Fang^1^; Zi-Yi Zhao^1^; Jin-Zhao Xie^1^; Jiao Chen^1^; Xiao-Yu Yin^1*^

This file contains following information:

**Supplementary Materials and Methods**

**Supplementary Figures**

**Supplementary Tables**

**Supplementary Materials and Methods**

**Western Blotting and Co-Immunoprecipitation analysis (Co-IP)**

Lysates were prepared in RIPA buffer with inhibitors, and 20 µg protein was separated by 10% SDS‑PAGE. Proteins were transferred to PVDF membranes, blocked with 5% milk/TBS‑T for 1 h, then incubated with primary antibody overnight at 4 °C. After washing, membranes were probed with HRP‑conjugated secondary antibody for 1 h and visualized by ECL.

Cells were lysed in IP Lysis Buffer (Thermo Fisher Scientific Inc.) supplemented with a protease inhibitor cocktail (Roche AG). Clarified lysates (15 000 × g, 15 min, 4 °C) were incubated overnight at 4 °C with target‐specific antibody or control IgG. Immune complexes were captured by adding Protein A/G magnetic beads (MCE, NJ, USA) for 2 h at 4 °C with gentle rotation. Beads were washed three times in lysis buffer, and bound proteins eluted in Laemmli buffer by heating at 95 °C for 5 min. Eluates were then analyzed by LC‑MS/MS or resolved by SDS‑PAGE for Western blot detection.

**Methylated RNA Immunoprecipitation (meRIP)**

Total RNA was isolated with Trizol Reagent (Invitrogen, USA) according to the manufacturer’s protocol. Polyadenylated RNA was enriched from 2 µg of total RNA using the Dynabeads mRNA Purification Kit (61006, Invitrogen, USA). The recovered mRNA was chemically fragmented and then subjected to immunoprecipitation with an anti‑m^6^A antibody utilizing the m^6^A Transcriptome Profiling Kit (C11051‑1, RIBOBIO, China). Both the input (non‑immunoprecipitated) and m^6^A‑enriched RNA fractions were converted into sequencing libraries and sequenced by Novogene (Beijing, China).

**RNA Immunoprecipitation (RIP)**

Gemcitabine-resistant PDAC cells (BxPC‑3 and CFPAC‑1) were harvested and lysed in RIP buffer supplemented with protease and RNase inhibitors. Lysates were cleared by centrifugation and incubated overnight at 4 °C with an anti‑IGF2BP1 antibody or control IgG, coupled to Protein A/G magnetic beads. Following extensive washes, bead‑bound complexes were treated with Proteinase K to digest proteins, and RNA was extracted using Trizol Reagent (Invitrogen, USA) according to the manufacturer’s instructions. The resulting RNA was split: one portion was used to generate libraries for high‑throughput sequencing (Novogene, Beijing, China), and the remainder was reverse‑transcribed and quantified by qPCR to validate enrichment of target transcripts.

**Liquid Chromatography Mass Spectrometry/Mass Spectrometry (LC-MS/MS)**

IGF2BP‑bound protein complexes were immunoprecipitated from BxPC‑3 cells in two independent co‑IP experiments. Eluted proteins were digested with trypsin, and resulting peptides were lyophilized, then reconstituted in 2% acetonitrile/0.1% formic acid. 20 µL aliquots were loaded onto a nanoViper C18 trap column (3 µm, 100 Å) and separated on an Easy nLC 1000 system (Thermo Fisher) using solvent A (98% water/2% acetonitrile/0.1% formic acid) and solvent B (98% acetonitrile/0.1% formic acid). After a 5 min trap at 100% A, peptides were eluted over 30 min with an 8–38% B gradient on an analytical C18 column (3 µm, 100 Å).

MS data were acquired on a Q Exactive mass spectrometer (Thermo Fisher) with a Nano Flex source at 275 °C and 1.9 kV spray voltage. Full-MS survey scans (250 ms) were followed by up to 20 higher‑energy collision dissociation MS/MS scans (50 ms) of precursors with charge states +2 to +4, employing a 25 s dynamic exclusion.

MS/MS data were analyzed using PEAKS Studio 8.5 (Bioinformatics Solutions Inc., Waterloo, Canada). The local false discovery rate at the peptide-spectrum match (PSM) level was set to 1.0% after searching against the Homo sapiens database with a maximum of two missed cleavages. The settings for variable modifications included oxidation (M), acetylation (protein N-term), deamidation (NQ), pyro-glu from E, and pyro-glu from Q, with fixed carbamidomethylation of cysteine. The mass tolerances for precursor and fragment ions were set to 10 ppm and 0.05 Da, respectively.

**Immunohistochemistry (IHC)**

Sections were deparaffinized, rehydrated through graded alcohols, and subjected to antigen retrieval in 10 mM Tris-EDTA buffer (pH 9.0) in a microwave. Endogenous peroxidase was quenched with 3% H₂O₂, and nonspecific sites blocked with 5% goat serum for 30 min. Sections were incubated overnight at 4 °C with primary antibody (e.g., anti‑IGF2BP1, 1:200), followed by 1 h with HRP‑conjugated secondary antibody. Signal was developed in DAB, counterstained with hematoxylin, dehydrated, cleared, and coverslipped. Slides were digitized and five random 400× fields per sample were quantified for positive staining using Image J. Staining intensity (SI) was assessed by multiplying the staining value (0, negative; 1, weak; 2, mild; 3, strong) by the percentage of stained cells (0, 0%; 1, 1-25%; 2, 26-75%; 3, 76-100%).

**Multiplex Immunofluorescence (mIF)**

Prior to immunofluorescence staining, hematoxylin and eosin (H&E) staining was performed on serial sections of each needle biopsy specimen. Tumor regions were independently identified by two experienced clinical pathologists based on classic histopathological features of PDAC, including irregular ductal structures, nuclear pleomorphism, and desmoplastic stroma. Regions exhibiting acinar morphology or other non-neoplastic characteristics were excluded. Only areas fulfilling the diagnostic criteria for PDAC were selected for immunofluorescence analysis and quantification.

Multiplex immunofluorescence (mIF) analysis was conducted using the PANO 4-plex IHC kit (Panovue, Beijing, China). Staining intensity (SI) was assessed by multiplying the staining value (0, negative; 1, weak; 2, mild; 3, strong) by the percentage of stained cells (0, 0%; 1, 1-25%; 2, 26-75%; 3, 76-100%).

For quantitative analysis, five representative high-power fields (HPFs) were randomly selected from the confirmed tumor regions of each specimen. Fluorescence intensity scoring was performed independently by two investigators in a blinded manner, without knowledge of the clinical response category. The final score for each sample was calculated as the average of the selected fields. Representative images shown in the figures were selected from fields that best reflected the overall staining pattern and were consistent with the quantitative results.

**Immunofluorescence**

Cells grown on confocal microplate were fixed with 4% paraformaldehyde for 15min at room temperature, followed by blocking with 5% bovine serum albumin (BSA) containing 0.1% Tween-20 for 1h at room temperature. The cells were then incubated with primary antibodies diluted in blocking buffer overnight at 4°C in a humidified chamber. After washing three times with PBST (5min each), the cells were incubated with the appropriate fluorescent secondary antibodies for 1h at room temperature in the dark. Nuclei were counterstained with DAPI for 3min. Finally, coverslips were mounted with antifade mounting medium and imaged immediately or stored at 4°C protected from light until imaging.

Colocalization analysis was performed using ImageJ (Coloc2 plugin), and Pearson’s correlation coefficients were calculated.

**Combination Index (CI) Analysis**

All combination-index (CI) analyses were performed in CompuSyn software (ComboSyn, Inc.). CI values were calculated according to the following equation:

$$CI=\frac{Dose of Agent A in Combination}{Dose of Agent A Alone}+\frac{Dose of Agent B in Combination}{Dose of Agent B Alone}$$

The CI value quantitatively measures the combined effect of two agents, indicating whether they act synergistically (CI < 1), additively (CI = 1), or antagonistically (CI > 1).

**Supplementary Figures**

**FigS1. IGF2BP1 is highly expressed in gemcitabine-resistant PDAC**

A. The IC_50_ value of wild-type (WT) and gemcitabine-resistant (GR) BxPC-3 cells;
B. The IC_50_ value of wild-type (WT) and gemcitabine-resistant (GR) CFPAC-1 cells;
C. Elevated IGF2BP1 expression in PDAC tumor tissues (n=146) compared to normal pancreatic tissues (n=170) (TCGA and GTEx datasets); two-tailed Student’s t-test for significance;
D. Kaplan-Meier survival curves of PDAC patients stratified by IGF2BP1 expression levels (TCGA datasets);

Data are presented as mean ± standard deviation (SD). *p < 0.05; **p < 0.01; ***p < 0.001.

**FigS2.** **IGF2BP1 is associated with gemcitabine resistance in PDAC both *in vitro* and *in vivo***

A. Effects of IGF2BP1 knockdown and overexpression on IC_50_ values of gemcitabine in CFPAC-1/GR cells;
B. Effects of IGF2BP1 knockdown and overexpression on cell proliferation in CFPAC-1/GR cells treated with 1000nM gemcitabine;
C. Effects of IGF2BP1 knockdown and overexpression on colony formation in CFPAC-1/GR cells treated with 500 nM gemcitabine; representative images (left) and quantification (right).
D. Mice harboring xenografts derived from IGF2BP1 knockdown and overexpressing CFPAC-1/GR cells were treated with gemcitabine (25mg/kg, twice a week, intraperitoneally, n=5 per group). Images of dissected tumors (left). Tumor weights (one-way ANOVA followed by Tukey’s post hoc test), tumor growth curves (repeated-measures ANOVA), and mouse weights across groups (right);
E. Representative images of IHC staining for Ki67 in xenografts from each group (n=5). Scale Bars, 200μm (top) and 50μm (bottom). Quantification of Ki67-positive cells was analyzed using one-way ANOVA followed by Tukey’s post hoc test.

Statistical significance for panels A–C was assessed using two-tailed Student’s t-test or one-way ANOVA followed by Dunnett’s post hoc test, as appropriate. Data are presented as mean ± standard deviation (SD). *p < 0.05; **p < 0.01; ***p < 0.001.

**FigS3. IGF2BP1-Mediated m^6^A Modification Sustains FTH1 mRNA Expression**

A. RIP-qPCR analysis using an anti-IGF2BP1 antibody or IgG control demonstrating the direct interaction between IGF2BP1 and FTH1 mRNA in CFPAC-1/GR cells;
B. Quantitative analysis of FTH1 mRNA expression levels following IGF2BP1 knockdown or overexpression in CFPAC-1/GR cells;
C. IGF2BP1-knockdown CFPAC-1/GR cells were treated with 5 μg/ml actinomycin D (ActD) to inhibit transcription, and the decay rate of FTH1 mRNAs was measured by qPCR.
D. Dual-luciferase reporter assay showing that mutation of the FTH1 m^6^A site significantly impaired IGF2BP1-mediated regulation of FTH1 in CFPAC-1/GR cells.
E. RIP-qPCR analysis demonstrating that mutation of the FTH1 m^6^A site markedly reduced the binding affinity of IGF2BP1 to FTH1 transcripts in CFPAC-1/GR cells.
F. Actinomycin D (ActD) chase assay showing that mutation of the FTH1 m^6^A site significantly decreased FTH1 mRNA stability in CFPAC-1/GR cells; one-way ANOVA with Tukey’s post hoc test for significance.
G. IC_50_ analysis showing that mutation of the FTH1 m^6^A site significantly increased the sensitivity of PDAC cells to gemcitabine in CFPAC-1/GR cells;
H. Multiplex immunofluorescence (mIF) staining of IGF2BP1 (green), FTH1 (red), and nuclei (DAPI, blue) in gemcitabine-resistant and gemcitabine-sensitive PDAC needle biopsy samples obtained prior to chemotherapy (n=20). Scale Bars, 100μm.

Statistical significance for panels A–C was assessed using one-way ANOVA with Dunnett’s post hoc test, as appropriate. Statistical significance for panels D–E was assessed using two-tailed Student’s t-test. Data are presented as mean ± standard deviation (SD). *p < 0.05; **p < 0.01; ***p < 0.001.

**FigS4. Stress Granule Formation by G3BP1 and IGF2BP1 Inhibits FTH1 mRNA Degradation**

qPCR analysis showing that knockdown of G3BP1 reverses the effects of IGF2BP1 depletion or overexpression on FTH1 mRNA levels in CFPAC-1/GR cells, two-tailed Student’s t-test or one-way ANOVA with Dunnett's test for significance.

Data are presented as mean ± standard deviation (SD). *p < 0.05; **p < 0.01; ***p < 0.001.

**FigS5. The IGF2BP1-FTH1 Axis Regulates Iron Homeostasis and Inhibits Ferroptosis**

A. Gemcitabine increases intracellular ROS levels in PDAC cells.
B. Quantification of lipid peroxidation (LPO) levels showing that IGF2BP1 knockdown increased, whereas IGF2BP1 overexpression decreased, intracellular LPO under gemcitabine treatment; these effects were reversed by reciprocal modulation of FTH1.
C. Quantitative analysis showing that, compared with the wild-type FTH1 rescue group, cells expressing m^6^A-mutant FTH1 exhibited significantly elevated LPO levels under gemcitabine treatment.
D. Effects of IGF2BP1 knockdown combined with the ferroptosis inhibitors Ferrostatin-1 (Fer-1) and deferoxamine (DFO), or the apoptosis inhibitor Z-VAD-FMK, on cell viability in CFPAC-1/GR cells treated with gemcitabine.
E. Quantification of intracellular Fe²⁺ accumulation and lipid peroxidation detected by Fluorescent probes (FerroOrange and Liperfluo), showing that IGF2BP1 knockdown elevates, and overexpression reduces, intracellular Fe²⁺ and LPO levels (n = 5 independent experiments), Scale Bars, 100μm.
F. Representative images of IHC staining for IGF2BP1, FTH1, 4-HNE and GPX4 in xenografts from each group (n=5). Scale Bars, 200μm. Statistical analysis of IGF2BP1, FTH1, 4-HNE and GPX4 expression in xenografts from each group (n=5, right).

Statistical significance for panels A–F was assessed using one-way ANOVA with Tukey’s post hoc test, as appropriate. Data are presented as mean ± standard deviation (SD). *p < 0.05; **p < 0.01; ***p < 0.001.

**FigS6. Targeting IGF2BP1 sensitizes PDAC to Gemcitabine**

A. Combination Index (CI) depicting the synergistic effect of gemcitabine and BTYNB on CFPAC-1/GR cells;
B. Dose-response curves illustrating the individual and combined treatment effects of gemcitabine and BTYNB on CFPAC-1/GR cells;
C. Cell growth curves of CFPAC-1/GR cells treated with PBS, BTYNB, gemcitabine, or the combination of gemcitabine and BTYNB;
D. Colony formation abilities revealing the effects of PBS, BTYNB, gemcitabine, or the combination of gemcitabine and BTYNB on CFPAC-1/GR cells.
E. qPCR verification showing that BTYNB treatment significantly reduced FTH1 mRNA expression in CFPAC-1/GR cells.
F. Statistical analysis showing kidney function of mice in different treatment groups (n=5 per group).
G. Typical images of IHC staining for FTH1 in gemcitabine-resistant PDXs from different treatment groups (n=5 per group). Scale Bars, 625μm (top) and 200μm (bottom). The IHC score of FTH1 in gemcitabine-resistant PDXs from different treatment groups (right).

Statistical significance for panels C-G was assessed using one-way ANOVA with Tukey’s post hoc test, as appropriate. Data are presented as mean ± standard deviation (SD). *p < 0.05; **p < 0.01; ***p < 0.001.

**Supplementary Tables**

**Table S1. Sequences of Primers Used in this Study**

| Primer |  | 5’-3’ Sequence |
| --- | --- | --- |
| IGF2BP1 | F | GCGGCCAGTTCTTGGTCAA |
|  | R | TTGGGCACCGAATGTTCAATC |
| FTH1 | F | CCATGTCTTACTACTTTGACC |
|  | R | GTCTGGTTTCTTGATATCCTG |
| G3BP1 | F | CCCGACGATGTGCAGAAGAG |
|  | R | CCCAAGAAAACGTCCTCAAGTC |
| GAPDH | F | GGAGCGAGATCCCTCCAAAAT |
|  | R | GGCTGTTGTCATACTTCTCATGG |
| β-actin | F | CATGTACGTTGCTATCCAGGC |
|  | R | CTCCTTAATGTCACGCACGAT |
| FTH1(meRIP) | F | ACTACCACCAGGACTCAGAGG |
|  | R | CAGGTAAACGTAGGAGGCGT |

**Table S2. Sequences of shRNA Used in this Study**

| Names | 5’-3’ Sequence |
| --- | --- |
| shRNA IGF2BP1-1 | ACGCTTAGAGATTGAACATTC |
| shRNA IGF2BP1-2 | CTCCAAAGTTCGTATGGTTAT CTCGAG |
| shRNA FTH1-1 | CCTCGGGCTAATTTCCCCATA CTCGAG |
| shRNA FTH1-2 | GCTTTGAAGAACTTTGCCAAA CTCGAG |
| shRNA G3BP1-1 | TCTCGGAATAGTCTATTATAA |
| shRNA G3BP1-2 | CCTCAGAGAGATCAGAGAGTT |

**Table S3. Antibodies Used in this Study**

| Name of Antibody | Catalog Number | Application | Brand of Antibody |
| --- | --- | --- | --- |
| anti-GAPDH | 10494-1-AP | WB | Proteintech |
| anti-β-actin | 4970 | WB | Cell Signaling Technology |
| Anti-G3BP1 | 61559 | WB, IF | Cell Signaling Technology |
| anti-IGF2BP1 | 8482 | WB, IHC, mIF | Cell Signaling Technology |
| anti-IGF2BP1 | 166344 | IF | Santa Cruz |
| anti-Ki67 | ab156956 | IHC | Abcam |
| anti-FTH1 | 4393 | WB, IHC | Cell Signaling Technology |
| Goat Anti-Rabbit IgG | ab150077 | IF | Abcam |
| Goat Anti-Mouse IgG | ab6785 | IF | Abcam |
| anti-Flag M2 Mouse | F1804 | co-IP | Sigma-Aldrich |
| anti-Myc Mouse | M4439 | co-IP | Sigma-Aldrich |
| anti-rabbit IgG | 7071 | ChIP, RIP | Cell Signaling Technology |
| anti-mouse IgG | 7076 | ChIP, RIP | Cell Signaling Technology |
